# Refinement of dietary exposure assessment using origin-related scenarios

**DOI:** 10.1038/s41370-019-0117-6

**Published:** 2019-02-06

**Authors:** Carolin Fechner, Matthias Greiner, Helmut Heseker, Oliver Lindtner

**Affiliations:** 1 German Federal Institute for Risk Assessment, Max-Dohrn-Straße 8–10, 10589 Berlin, Germany; 2 University of Veterinary Medicine Hannover, Foundation, Hannover, Germany; 3 University Paderborn, Warburger Straße 100, 33098 Paderborn, Germany

**Keywords:** Dietary exposure, pesticides, metals

## Abstract

Global sourcing of food may lead to variability in concentrations of contaminants or pesticide residues. It would be important to incorporate origin influences in dietary exposure assessment. To characterise uncertainties, substance concentrations from GFM (German Food Monitoring), chosen based on the highest CV (coefficient of variation), and food consumption from NVS II (German National Nutrition Survey II) were combined in standard scenarios. Averages or higher percentiles of non-grouped concentrations were used. Additional origin-related scenarios used concentrations grouped by origin. For bromide in tomatoes the most conservative origin-related scenario for Italian tomatoes resulted in the highest exposure of 0.015 mg/d/kg BW. The impact of origin was not covered by the conservative standard scenario (0.006 mg/d/kg BW). For ethephon in pineapples and aluminium in kiwifruits, the highest intake estimates were obtained with the conservative standard scenario resulting in 0.895 μg/d/kg BW and 0.023 mg/week/kg BW, respectively. In these two cases, standard scenarios cover origin influences but the conservative origin-related scenario based on origins with higher concentrations identifies lower exposures of 0.835 μg/d/kg BW for ethephon from African pineapples and 0.014 mg/week/kg BW for aluminium from non-EU kiwifruits. Hence, the inclusion of origin information can refine exposure assessment.

## Introduction

Food supply is becoming more global, especially in the sourcing of raw materials and the food ingredients [1]. Regulations on food trade and labelling ensure consumer protection [2]. To guarantee an all-season availability of agricultural products, cross-country and cross-continental trade is intensified, which increases the complexity in food supply [3]. For dietary exposure assessment, as an important base for risk assessment [4], the relation between substance concentration and geographical food origin is of interest. For example, for cadmium in chocolate there is a relevance of origin shown [5, 6]. In major steps of food supply, different influences on substances and finally on dietary exposure are possible [7–9]. Conditions in agricultural production could influence substance concentration in foods, as well as factors like time, climate or contact materials which could be relevant in transport, storage and processing.

Mandatory origin information on foods allows the identification of the geographical primary production. In the European Union (EU), the indication of country of origin or place of provenance is mandatory, e.g., for unprocessed beef, as the place of birth has to be stated [10] as well as for fruits and vegetables because of marketing standards [11–13] and for some other food products. Additionally, voluntary origin information is permitted [14, 15]. The limited obligation to label the primary geographical food origin [14, 16, 17] is an obstacle for a refined dietary exposure assessment that should account for food origin.

This paper aims to use available data on substance concentrations grouped by geographical food origin to study the influence of origin on standard deterministic dietary exposure assessment. A refined approach for assessment strategies based on limited data is introduced, while considered case studies do not necessarily represent cases of existing health risks. The exposure assessment focusses on chronic intakes. In particular, it is investigated whether conservative assumptions of standard scenarios [4] could cover calculations of origin-specific scenarios. Information on food origin and its influence on substance concentrations is included in exposure assessment, while standard scenarios focus generally on average and high concentrations which are not origin specific. Based on case studies, we want to investigate possible origin influences in dietary exposure assessment.

## Materials and methods

### Substance concentrations and geographical food origin

GFM (German Food Monitoring) data, including projects between 2005 and 2015, were used to derive substance concentrations in the fresh weight of food in connection with origin information [18–20]. First, various available agricultural products with obligatory country of origin labelling were considered [11–13, 15]. These foods were checked for substances with a high percentage (>50%) of quantifiable concentrations and minimum 40 samples. For further considerations, the coefficient of variation (CV) as the ratio of standard deviation (SD) and mean was applied to select the substance with the highest variation (Table 1). Then, origin-related variability in concentrations was considered using means and boxplots per country. Finally, the following case studies had origin relations in substance concentrations and were selected according to the coding in GFM: [21, 22]

- Unprocessed, unpeeled tomatoes (code: 250301) and bromine containing fumigants calculated as bromide (following referred to as bromide, code: 3808008);
- Unprocessed, unpeeled pineapples (code: 290501) and ethephon (code: 3810008); and
- Unprocessed, peeled kiwifruits (code: 290513) and aluminium (code: 1813000).

**Table 1:** Variation of substance concentrations in food samples matching the predefined conditions of a minimum of 40 samples and more than 50% quantifiable concentrations

Food	Substance	*N*	Mean^a^ [mg/kg]	SD^a^ [mg/kg]	CV
Tomatoes	Bromide	714	1.3	3.1	2.5
Pineapples	Ethephon	213	0.2	0.4	2.1
	Triadimenol	358	0.2	0.3	1.4
	Triadimefon	333	0.1	0.1	1.1
	Sum of Triadimefon and Triadimenol	358	0.3	0.3	1.2
Kiwifruits	Aluminium	163	1.0	1.7	1.8
	Manganese	47	0.5	0.2	0.5
	Zinc	207	1.1	0.4	0.4
	Copper	207	1.5	0.5	0.3

Data basis: GFM (German Food Monitoring) 2005–2015

*SD* standard deviation, *CV* coefficient of variation as the ratio of SD and mean

^a^Calculated by modified lower bound approach (MLB)

Tomatoes, pineapples and kiwifruits are supplied by different countries [3, 23–25] Origin information from GFM, mostly country of origin but also continents [26], was used to connect substance concentrations in food with geographical origins which is displayed in Table 2. Unspecific information like *without declaration*, *unexplained* or *unknown foreign country* was summarised.

**Table 2:** Substance concentrations in food samples by geographical origin

Case study: substance–food	Geographical origin	*N*	*N* quantifiable	Mean^a^	SD^a^
				mg/kg	
Bromide–Tomatoes	Senegal	4	2	0.2	–
	Bulgaria	1	0	0.2	–
	Germany	145	74	0.3	0.5
	Turkey	5	4	0.4	0.3
	Not specified^b^	23	17	0.5	0.5
	France, incl. Corsica	17	13	0.6	0.7
	Malta	2	2	0.7	–
	The Netherlands	232	176	0.8	0.8
	Belgium	40	27	0.8	0.9
	Spain	177	153	1.2	0.9
	Israel	12	12	2.1	1.5
	Morocco	16	13	2.2	4.3
	Italy	40	38	8.6	9.7
Ethephon–Pineapples	South Africa	2	0	0.0	–
	Turkey	1	1	0.0	–
	Panama	8	8	0.1	0.1
	Costa Rica	156	132	0.1	0.2
	Cote d’Ivoire	3	2	0.2	–
	Not specified^b^	12	11	0.2	0.2
	Africa	1	1	0.2	–
	Ecuador, incl. Galapagos Islands	8	8	0.2	0.4
	Honduras	4	4	0.3	0.3
	Cameroon	2	2	0.4	–
	Ghana	14	14	0.8	0.6
	Mauritius	2	2	2.6	–
Aluminium–Kiwifruits	Not specified^b^	6	2	0.2	0.3
	Greece	20	12	0.3	0.3
	France, incl. Corsica	4	3	0.4	0.6
	Italy	96	63	0.5	1.0
	Spain	1	1	0.6	–
	Chile	7	7	2.3	1.4
	New Zealand	29	24	3.0	2.8

Data basis: GFM (German Food Monitoring) 2005–2015

*SD* standard deviation

^a^Calculated by modified lower bound approach (MLB)

^b^“Without declaration”, “unexplained” and “unknown foreign country” summarised

### Plausibility of available information on geographical food origin

To evaluate the credibility of specific origin information in GFM for further grouping of substance concentrations, declarations were compared with FAO (Food and Agriculture Organization of the United Nations) data on the cultivation of appropriate crops [27]. This is important because the annual GFM reports declare that especially the stated origin Germany does not necessarily correspond to the country of origin but to the place of processing or packaging [28]. Countries stated in GFM were selected in FAO data and were then checked for existing crop yields within the monitoring years viewed in GFM. An origin stated in GFM was evaluated as plausible if it was available as a cultivation area in FAO data. If a continent instead of a specific country was given in GFM, FAO data were checked for countries within this continent.

### Dealing with non-detectable and non-quantifiable substance concentrations

A modified lower bound approach (MLB) was applied for the determination of statistical parameters for substance concentrations, as for some samples concentrations were not quantifiable but should be included in calculations. Therefore, a replacement of non-detects with zero and non-quantified values with the limit of detection (LOD) was realised [29]. Additionally, an upper bound approach (UB) was performed substituting non-detects with the LOD and non-quantified values with the limit of quantification (LOQ) [29]. Statistical parameters derived by MLB were used for all further considerations and differences to UB were discussed later on.

### Annual and seasonal differences in substance concentrations

GFM data of several years were used for the consideration of origin-related substance concentrations in food. Mean substance concentrations per year were calculated to examine inter-annual variations in concentrations. Because of non-normal distributed concentrations, testing for significant differences between years was performed using the non-parametric Kruskal–Wallis test in SPSS version 21 with a significance level of *P* ≤ 0.05. The season could also have influences on substance concentrations [30]. Seasonal effects were considered with scatterplots showing a graphical distribution of substance concentrations per month. GFM data were checked additionally for countries of origin per month to evaluate the variation in supply during the year. The same analysis was done using BLE (Federal Office fo`r Agriculture and Food) data between 2013 and 2015 [31].

### Grouping of substance concentrations by geographical origin

Data of Table 2 with classified case studies, which had substance concentrations related to geographical origins, were used for grouping to compare mean concentrations from specific regions with the situation of all samples. *Origin A* with lower mean concentrations and *origin B* with higher mean concentrations were defined as sub-divisions of *all samples*. In this way similar mean concentrations from single origins of sample numbers lower than 20 were summarised to groups of larger regions (e.g., collateral countries, continents) and more than 20 samples to determine 95th percentile (P95) and other statistical parameters. Implausible origin information was excluded from grouping.

Mean concentrations of *all samples*, *origin A* and *origin B* were tested for significant differences using SPSS version 21. The application of the Kolmogorov–Smirnov test showed normal distributed concentrations for *origin B* in all case studies; for the other groups, no normal distribution was attested. Therefore, the non-parametric Kruskal–Wallis test in combination with the post-hoc Dunn–Bonferoni test were used for multiple mean comparisons between *all samples*, *origin A* and *origin B* with a significance level of *P* ≤ 0.05.

### Food consumption

The dietary history interview from the German NVS II (National Nutrition Survey II) was used, giving information on average long-term food consumption [32]. The dietary history was a retrospective request over 4 weeks and documented the frequency and quantity of foods and beverages usually consumed by 15,371 participants aged between 14 and 80 years as a representative sample for the German population (Table 3) [32]. Only data of consumers were taken for further investigations. To gain representative consumption data for fresh or self-prepared tomatoes, pineapples or kiwifruits, a disaggregated data version was used. Therefore, recipe codes *xy* were disaggregated previously by BfR (German Federal Institute for Risk Assessment) using BLS (Bundeslebensmittelschlüssel) recipes version II.4. The percentage of consumers is quite high for unprocessed tomatoes (97%), while pineapples and kiwifruits are consumed by less than 50% of the sampled population (18% and 30%) (Table 3).

**Table 3:** Food consumption in Germany for tomatoes, pineapples and kiwifruits (consumers only)

Food consumption	Tomatoes	Pineapples	Kiwifruits
Mean [g/d/kg BW]	0.6	0.3	0.2
P95 [g/d/kg BW]	1.7	1.1	0.7
Number of consumers	14,981	2757	4660
Number of participants	15,371	15,371	15,371
Percentage consumers [% per month]	97	18	30

Data basis: NVS II (German National Nutrition Survey II), dietary history interview (recorded frequency and quantity of foods and beverages usually consumed retrospectively over 4 weeks), participants aged between 14 and 80 years

*P95* 95th percentile, *BW* body weight

### Chronic dietary exposure assessment

**Fig. 1:**
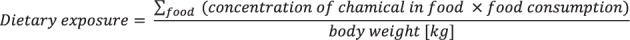
Calculation of dietary exposure (acute and chronic) [29]

**Fig. 2:**
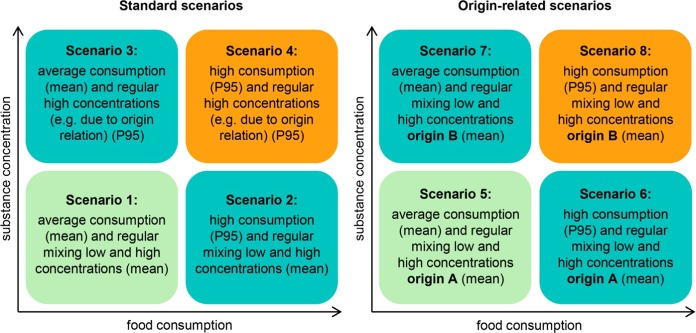
Standard scenarios for deterministic dietary exposure assessment (according to Sarvan et al. [4]) (left) and origin-related scenarios (right). Same colour shows corresponding scenarios (standard (1–4) and origin-related (5–8)). While standard scenarios use P95 (95th percentile) for high concentrations, origin-related scenarios use mean concentrations of different origins

Chronic dietary exposure assessment was performed using a general model (Fig. 1) [29]. If several foods are modelled, they are summed up on the individual level. Uptake factors for substances inside the body are assumed to be one. In a deterministic approach, the combination of different distribution parameters of substance concentration with food consumption was based on four standard scenarios based on concentrations of all samples (Fig. 2) [4]. These were extended by four origin-related scenarios regarding grouped origin-specific substance concentrations as subsets of all samples from GFM to compare standard exposure with origin-related situations (Fig. 2). Distribution parameters are mean and P95 to model mean consumption or mixing concentrations and high consumption or high concentrations. A chronic modelling of substance intake made it possible to investigate long-term influences and to pay attention to more or less stable food supply.

To show the differences in exposure, ratios of each standard scenario to an appropriate origin-related scenario were derived using the same amount of food consumption (mean or P95). Grouped mean concentrations from *origin A* were compared with mean concentrations of *all samples*, as well as mean concentrations from *origin B* with high concentrations (P95) of *all samples*. In this way an appropriate origin-related scenario is constructed for each standard scenario which is displayed with same colours in Figs. 2 and  4. Origin-related scenarios used mean concentrations only because the inclusion of P95 concentrations might be too conservative, as assumed consumer behaviour is modelled without having additional survey data.

The calculated exposures for the selected substances were compared with appropriate health-based guidance values, especially acceptable daily intake (ADI) and tolerable weekly intake (TWI) [33].

### Statistics

Statistical analyses were carried out using SPSS version 21. SD was only provided for groups of at least four quantified samples and P95 was only calculated for groups of at least 20 quantified samples using PTILE within the CTABLES command. Microsoft Excel 2010 was used for exposure assessment and graphical depiction.

## Results

### Plausibility of available information on geographical food origin

For samples of tomatoes and kiwifruits, there is no implausible origin information in GFM compared to FAO data, but there are some unspecified data (<5%) which cannot be matched to specific regions (Table 4). Considering pineapples, 5.6% of the origin classifications are not further specified (Table 4). Only for one sample from Turkey (Table 2), no data are available from FAO, which means pineapples are not cultivated there. Therefore, this origin is classified implausible. GFM data for tomatoes, kiwifruits and pineapples are suitable for further grouping of substance concentrations by origin.

**Table 4:** Plausibility of available origin information for food samples

Information on geographical origin	Tomatoes		Pineapples		Kiwifruits	
	*N*	%	*N*	%	*N*	%
Plausible	691	96.8	200	93.9	157	96.3
Not specified	23	3.2	12	5.6	6	3.7
implausible	0	0.0	1	0.5	0	0.0
Total	714	100.0	213	100.0	163	100.0

Data from GFM (German Food Monitoring) 2005–2015 compared with FAO (Food and Agriculture Organization of the United Nations) data on crops

### Annual and seasonal differences in substance concentrations

There are no obvious temporal trends in ethephon concentrations in pineapples between the two years considered as no significant differences were found. For aluminium in kiwifruits concentration trends cannot be evaluated, as only data from 2010 are available (Table 5). For bromide in tomatoes, there are four monitoring years available. In 2010, the mean concentration is slightly lower (0.9 mg/kg) than in the other years (Table 5). As the mean concentration is higher again (1.0 mg/kg) in 2013, there is no obvious time shift for decreasing concentrations visible and, hence, no significant differences were found.

**Table 5:** Substance concentrations in food samples by monitoring years

Case study		Available concentration data			
Substance	Food	Year	*N*	Mean^a^	SD^a^
				mg/kg	
Bromide	Tomatoes	2005	213	1.4	4.0
		2007	177	1.6	3.5
		2010	150	0.9	1.8
		2013	174	1.0	2.1
Ethephon	Pineapples	2010	110	0.2	0.6
		2013	103	0.2	0.2
Aluminium	Kiwifruits	2010	163	1.0	1.7

Data basis: GFM (German Food Monitoring) 2005–2015

*SD* standard deviation

No significant differences between the mean concentrations per case study were found according to Kruskal–Wallis test (*P* ≤ 0.05)

^a^Calculated by modified lower bound approach (MLB)

**Fig. 3:**
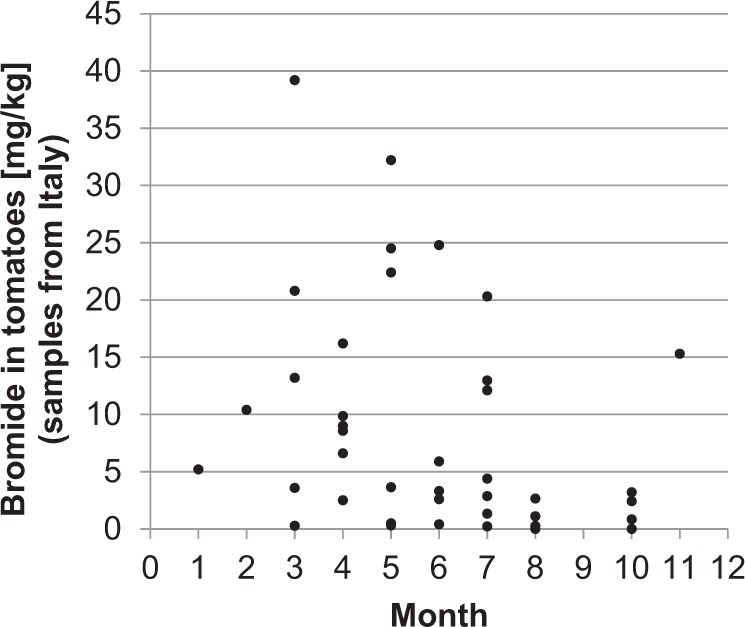
Bromide in Italian tomatoes by months (modified lower bound approach (MLB)). Based on GFM (German Food Monitoring) 2005–2015

Seasonal considerations of the case studies do not show higher substance concentrations in a certain period, i.e., single higher concentrations appear but are more or less evenly distributed over the course of the year and there are low sample numbers for some months. This can be seen in the example of bromide in tomatoes for samples from Italy (Fig. 3).

Regarding the seasonal supply during the course of the year, tomatoes are sourced from Italy, the Netherlands and Spain the whole year according to GFM and BLE data, other countries are present in parts of the year. GFM data for pineapples show an all-year supply from America and an additional supply from Africa at the beginning and in the middle of the year (BLE data were not available). According to GFM and BLE kiwifruits are supplied from EU countries to Germany most of the year with a gap around August; non-EU countries are mostly relevant between May and December.

### Grouped substance concentrations by geographical origin

Table 6 shows substance concentrations for *all samples* and grouped by geographical origin (*origin A* for lower concentrations and *origin B* for higher concentrations). There are statistically significant differences in mean concentrations between *origin B* and *all samples*, as well as between *origin B* and *origin A* which shows an effective grouping of higher concentrations (*origin B*) for the use in comparative considerations in exposure assessment.

**Table 6:** Substance concentrations of food samples grouped by geographical origin

Case study: substance–food	Grouped geographical origin*	*N*	*N* quantifiable	Mean**	SD**	P95**
				mg/kg		
Bromide–Tomatoes	*All samples*	714	531 (74%)	1.3^a^	3.1	3.2
	*Origin A*: all regions except Italy	674	493 (73%)	0.8^a^	1.1	2.5
	*Origin B*: Italy	40	38 (95%)	8.6^b^	9.7	28.5
Ethephon–Pineapples	*All samples*	213	185 (87%)	0.2^a^	0.4	0.8
	*Origin A*: America	176	152 (86%)	0.1^a^	0.2	0.6
	*Origin B*: Africa	24	21 (88%)	0.8^b^	1.0	1.8
Aluminium–Kiwifruits	*All samples*	163	112 (69%)	1.0^a^	1.7	4.7
	*Origin A*: EU	121	79 (65%)	0.5^a^	0.9	1.5
	*Origin B*: non-EU	36	31 (86%)	2.9^b^	2.6	9.6

Data basis: GFM (German Food Monitoring) 2005–2015

*EU* European Union, *SD* standard deviation, *P95* 95th percentile

*All samples: all available substance concentrations; *Origin A*: origin-related lower mean substance concentrations out of all samples; *Origin B*: origin-related higher mean substance concentrations out of all samples

**Calculated by modified lower bound approach (MLB)

^a,b^Significant differences between the mean concentrations per case study according to Kruskal–Wallis test (*P* ≤ 0.05) are shown by different index letters *a* and *b*. For example, significant differences of bromide concentrations in tomatoes occur between *origin B* and *origin A* as well as *origin B* and *all samples* but not between *all samples* and *origin A*

For bromide in tomatoes, Italy is classified as *origin B*, and *origin A* summarises all other regions and unspecific origins because lower concentrations cannot be connected to a specific geographical region (Table 6). This case study shows the largest origin-related differences, as the mean bromide concentration of 8.6 mg/kg of *origin B* is 6.9-fold higher than the mean concentration of *all samples* and 10.5-fold higher than the mean concentration from *origin A*.

Concerning ethephon in pineapples, the origin grouping is on a continental level; higher concentrations are attributed to fruits from Africa (*origin B*), while lower values are related to America (*origin A*) (Table 6). In the case of kiwifruits, higher aluminium concentrations are connected to fruits originating from non-EU countries (*origin B*) in comparison to kiwifruits from European states (*origin A*).

### Exposure

**Fig. 4:**
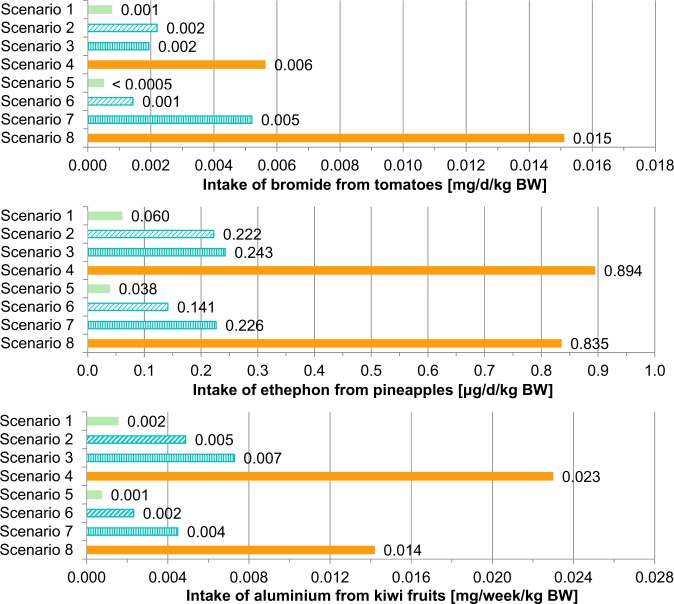
Chronic intake of substances from food. Calculations based on GFM (German Food Monitoring) 2005–2015 and NVS II (German National Nutrition Survey II) dietary history interview. Same colour and pattern show corresponding scenarios (standard (1–4) and origin-related (5–8)). While standard scenarios use P95 (95th percentile) for high concentrations, origin-related scenarios use mean concentrations of different origins. BW body weight

For bromide from tomatoes, a considerable origin influence on exposure is observed because high consumption and regular mixing of low and high concentrations from *origin B* (scenario 8) result in the highest intake estimate of 0.015 mg/d/kg body weight (BW) in comparison to high consumption and regular high concentrations of *all samples* (scenario 4) where the calculated exposure amounts to 0.006 mg/d/kg BW (Fig. 4). The exposure derived from *origin B* (Italy, Fig. 4) with defined higher bromide concentrations in tomatoes (scenario 8: P95 consumption, mean concentration of *origin B*) is 2.7-fold higher than the calculated value from the most conservative standard scenario 4 (P95 consumption, P95 concentration of *all samples*) (Table 7). The same ratio applies between scenarios 7 (mean consumption, mean concentration of *origin B*) and 3 (mean consumption, P95 concentration of *all samples*). The comparison of scenarios 5 (mean consumption, mean concentration of *origin A*) and 1 (mean consumption, mean concentration of *all samples*), as well as scenarios 6 (P95 consumption, mean concentration of *origin A*) and 2 (P95 consumption, mean concentration of *all samples*), shows that bromide exposure from *origin A* represents 65% of the calculated intake via unspecific mean concentrations of *all samples* (Table 7). For bromide from tomatoes, the highest intake estimate of 0.015 mg/d/kg (scenario 8: P95 consumption, mean concentration of *origin B*) represents 1.5% of the ADI which amounts to 1 mg/d/kg BW [34]. All other exposure scenarios result in intake estimates which represent less than 1% of the ADI.

**Table 7:** Ratio of standard exposure scenarios to corresponding origin-related exposure scenarios

Case study: substance–food	Ratio of exposure from different corresponding scenarios			
	Scenarios 8:4	Scenarios 7:3	Scenarios 6:2	Scenarios 5:1
Bromide from tomatoes	2.69	2.69	0.65	0.65
Ethephon from pineapples	0.93	0.93	0.64	0.64
Aluminium from kiwifruits	0.62	0.62	0.47	0.47

Each standard exposure scenario focusses concentrations of *all samples* (mean or P95 (95th percentile)) while the corresponding origin-related scenario focusses mean concentrations from grouped *origin A* or *origin B* to compare standard situations with origin-related situations

For the intake of ethephon from pineapples and aluminium from kiwifruits, an origin influence is given as well but lower than observed for bromide from tomatoes. High consumption and regular high concentrations of *all samples* (scenario 4) result in highest intake estimates (Fig. 4). In all direct comparisons, dietary exposure from origin-related scenarios is lower than intake estimates of corresponding standard scenarios displayed by ratios below 1 (Fig. 4, Table 7). This means that standard scenarios with high concentrations cover the regional influence shown in origin-related scenarios. For ethephon, an ADI of 0.03 mg/d/kg BW is given and for aluminium a TWI of 1 mg/week/kg BW is fixed [35, 36]. For ethephon from pineapples, the highest intake estimate (scenario 4: P95 consumption, P95 concentration of *all samples*) represents 3% of the related ADI. It is similar for aluminium from kiwifruits, as the highest exposure (scenario 4: P95 consumption, P95 concentration of *all samples*) represents 2.3% of the TWI.

## Discussion

Substance concentrations and geographical food origins were derived using GFM data. Groups of 20 samples are sufficient to determine a P95 which is lower than the maximum but it is not statistically robust. Therefore, only P95 of all samples per case study was used for exposure calculations. It is worthwhile to analyse origin influences on concentrations in cases where country of origin information is available. As there is no mandatory origin labelling for most of the processed foods, only agricultural products are investigated in this study. On the one hand, GFM data are not representative for the market composition because samples are not taken by origin and not every supplied cultivation area is represented in the existing proportion [19]. However, on the other hand, the dataset provides much information on samples taken from the German market, and hence is suited to identify origin-related concentrations in food and gives first insights into origin connections. The food analysis method is important for the interpretation of concentrations. In the case of ethephon in pineapples, the analysis is carried out for the fruit including the skin [37]. This may result in higher concentrations than actually consumed, but this is not relevant, as the analysis method is applied to all concentration data for pineapples. No processing factor is applied for peeling to avoid further uncertainties and to follow a conservative approach.

Information available on geographical food origin is checked for plausibility using FAO data. Some uncertainty in the origin information of GFM is left because the comparison cannot reveal unintended wrong reporting by surveillance authorities e.g. importers or packagers instead of the primary origin [28]. For example, 20% of the tomato samples considered have German origin, which could be related to the sampling strategy but also to wrong declaration or reporting. As Italy is a great distributor of tomatoes [25], the same applies to Italian tomato samples which show a significantly higher mean bromide concentration (Table 6). They could be packaged in Italy but produced elsewhere, hiding an unknown supply chain.

To deal with non-detectable and non-quantifiable substance concentrations, MLB and UB are used. As some laboratories have higher LOD or LOQ than actual measured concentrations, differences in maxima or P95 between MLB and UB can arise. This is the case for some origins with low sample numbers (tomatoes from Senegal (N = 4), pineapples from South Africa (N = 2) and kiwifruits from France (N = 4) (Table 2)). After grouping *origin A* for aluminium in kiwifruits (N = 121) (Table 6) shows 1.5 mg/kg (MLB) and 2.5 mg/kg (UB) as P95. The exclusion of the laboratory with higher LOD and LOQ respectively results in a loss of 15 samples (thereof 10 non-detectable), a unified P95 of 1.6 mg/kg for grouped *origin A* and no further differences in maxima or P95 between MLB and UB for other origins. Finally, no laboratory data is excluded from analysis, as evaluations are only done for MLB. Additionally, the UB represents more conservative assumptions [29].

Substance concentrations were grouped by geographical origin. Significant higher concentrations are grouped in *origin B* (Table 6). The sample number of *origin B* is smaller in comparison to *origin A* and more influenced by higher values. Results give insights into origin relations of concentrations, but for more comprehensive details, a representative sampling by origin is required. For tomatoes, a significantly higher mean bromide concentration from Italy (*origin B*) in comparison to other countries (*origin A*) is observed (Table 6). As *origin A* contains unspecific origins, some Italian samples could also be included. Methyl bromide was used in plant protection until the global phase-out in 2015 [38, 39]. Europe banned the usage in plant protection in 2010, but critical uses, where no alternative substances are available, or applications in the case of emergencies are still allowed [38, 40]. Studies on pesticide residues in Europe show origin-related different bromide concentrations in fruits and vegetables because of different methyl bromide use [41–43]. Natural bromide concentrations are higher in coastal regions because of the transfer of bromide contained in sea water to air, soil and ground water [44–46]. In Italy, tomatoes are intensively cultivated but methyl bromide is replaced by alternatives [47, 48]. This suggests that higher bromide concentrations in Italian tomatoes could be related to natural sources like the Mediterranean Sea.

For ethephon in pineapples, a significantly higher mean concentration from Africa than in American samples is observed (Table 6). The plant growth regulator ethephon is commonly present in European food samples [43]. According to GAP (good agricultural practice) the use is different in various producing countries depending on crops, quantity of addition and time intervals until harvest [49]. Large amounts of pineapples are supplied by the producing countries Ghana and Costa Rica which represent the main providers from Africa and America. However, there is a shift to cultivars from Costa Rica [23, 50, 51]. Investigations of the GAP in Ghana show partly too high ethephon concentrations in pineapples for export to the EU [52]. More pineapples are supplied by Costa Rica [51].

The mean aluminium concentration is significantly higher in non-EU kiwifruits in comparison to fruits from Europe (Table 6). Aluminium is an environmental contaminant and found in fruits; higher amounts also occur in processed food because of additives or packaging [36, 53, 54]. Plants can absorb aluminium via their roots [55–57]. Aluminium is more readily available for plants in acidic soils which is often the case in Chile [58]. This could be an explanation for higher concentrations in kiwifruits from Chile and countries having similar conditions (Table 2). A relation of aluminium concentration and geographical origin is, for example, observed in olive oil and coffee [59, 60] and for minerals in fruits [61]. New Zealand and Italy are the biggest kiwifruit exporters [3, 25]. A total diet study (TDS) of Australia (mainly supplied by New Zealand [3, 25]) and New Zealand shows a mean aluminium concentration of 2.2 mg/kg in kiwifruits [62] which is similar to the mean aluminium concentration of 2.9 mg/kg for non-EU kiwifruits in the current study (Table 6). Furthermore, transport conditions could play a role because kiwifruits are shipped, transferred repeatedly and partly packaged in cardboard which is produced using aluminium sulphate [63].

Exposures calculated for different scenarios show the influence of assumptions and uncertainties in the approach. Focussing on only one food origin as a possible consumer behaviour is used as the base of origin-related scenarios to model a long-term consumption of lower (*origin A*) or higher substance concentrations (*origin B*). In this way, conscious consumer decisions for foods from specific regions, but also the possible preference of brands, varieties or selling points as unconscious influences on the choice of food origin, are integrated. More knowledge on consumer habits in relation to food origin is required for a refined scenario construction. The comparison of intake estimates derived from standard scenarios and origin-related scenarios shows a clearly possible refinement in exposure assessment integrating data on food origin (Fig. 4).

Health-based guidance values are used to assess the risk of substances to humans comparing them with the calculated exposure [33]. For the bromide ion, the ADI is set to 1 mg/d/kg BW by the FAO [34], but it is not accepted by EFSA (European Food Safety Authority) [43]. The EFSA proposes an ADI for methyl bromide of 0.001 mg/d/kg BW [64]. The ADI for bromide ion is used to assess the results of the current study because bromide is the analysed substance in GFM. This is supported by former investigations on dietary exposure which used the bromide ADI for assessments as well [65, 66].

For bromide exposure from tomato consumption, the conservative standard scenario 4 results in 0.006 mg/day/kg BW which represents 0.6% of the bromide ADI. The conservative origin-related scenario 8 results in 0.015 mg/day/kg BW representing 1.5% of the ADI (Fig. 4). Other studies investigate the chronic total exposure to bromide reflecting to pesticide residues in fruits and vegetables. The EFSA consideration fixes 0.006 mg/day/kg BW for bromide in a lower bound approach (LB) [43] which corresponds to scenario 4. The LB of a Belgian study shows similar results with 0.1–1.0% of the ADI [65]. The bromide exposure from tomato derived from conservative scenarios in this study is similar to the total bromide exposure in other studies. A French investigation uses consumption data of children to calculate the ATMDI (adjusted theoretical maximum daily intake) and is more conservative assessing exposure with maximum residue levels of bromide which results in a 33.7% exploitation of the ADI with a mean total exposure and 67.5% of the ADI with P95 of total exposure [66]. This matches the calculations of this study, as tomato is just one source of bromide in nutrition. For bromide from tomato, on the one hand, it is not sufficient to focus on unspecific high concentrations (P95) of *all samples* because an underestimation of exposure is possible if geographical variability of concentrations is relevant to certain consumer groups. On the other hand, focussing on defined lower mean concentrations of *origin A* could help to prevent overestimation of exposure in relevant consumption situations.

The ethephon intake from pineapples results in 0.894 μg/day/kg BW (scenario 4) which represents 3.0% of the ADI and 0.835 μg/day/kg BW (scenario 8) representing 2.8% of the ADI (Fig. 4). The EFSA calculates an LB long-term total exposure of 0.4% of the ADI, the corresponding UB shows 2.0% [43]. Belgian scientists calculated a total exposure of 0.1–0.9% of the ADI using data on fruits and vegetables in an LB approach [65]. The demonstrated ethephon intake from pineapple in conservative scenarios of the current investigation is higher than the total exposure determined by EFSA [43] as well as by Belgian scientists [65]. A French study on the total exposure results in 18.5% of the ADI as mean total exposure and 37.4% of the ADI as P95 total exposure using ATMDI for children [66]. This matches the calculations of this study, as pineapples are only one source of ethephon intake.

Chronic dietary exposure assessment on aluminium from kiwifruits results in 2.3% of the TWI in scenario 4 (0.023 mg/week/kg BW) and 1.4% of the TWI in scenario 8 (0.014 mg/week/kg BW) (Fig. 4). According to the EFSA, the total aluminium exposure from food and water for adults amounts to 0.2–1.5 mg/week/kg BW [36]. Results of the 2nd French TDS show a mean exposure of adults of 0.28 mg/week/kg BW and a P95 exposure of 0.49 mg/week/kg BW [67]. The findings of the current study are in line with other investigations, as they are lower than the calculated total exposures, and kiwifruits are not the main source of aluminium and can only be a part of dietary exposure. As aluminium is ubiquitous in various food products [36], analysis of concentrations varying by origin could be important in a total dietary exposure assessment.

For ethephon from pineapples and aluminium from kiwifruits, according to our study, on the one hand, it is sufficient to focus on high consumption (P95) and unspecific high concentrations (P95) of *all samples* (scenario 4) because the influence of origins with defined higher concentrations (mean concentrations of *origin B*) combined with P95 consumption in scenario 8 is covered by an overestimation following conservative assumptions. There is a need to perform all standard scenarios to cover the possible origin influences. It is not enough to pay attention to mean consumption or mean concentrations of *all samples* only (scenarios 1–3) because scenario 8 exceeds these exposure estimates. On the other hand, an additional focus on origin-related scenarios could help to prevent overestimation of exposure and create refined approaches.

## Conclusion

Origin-related sampling and testing of food is required for those substances which are known to have a geographical component in the prevalence of contamination or level of contamination. The studies for bromide in tomatoes, ethephon in pineapples and aluminium in kiwifruits on the German market show that case-by-case evaluation is required and that conservative standard scenarios without considering food origin may underestimate or overestimate the exposure. Extended investigations would be required to clarify the reasons for regional differences and systematic case studies would be necessary to generalise coherences in food origin and exposure assessment. Access to industry self-control data could allow exposure and risk assessment to account for origin-specific scenarios. The extension of the country of origin labelling on processed foods, studies on consumer behaviour and preferences on food origin as well as on supply patterns to Germany and other countries could give additional valuable data for scenario construction. With knowledge of food origin, dietary exposure estimates can be refined and more informative origin-specific scenarios can be used. Future research could also address origin-specific scenarios in dietary exposure assessment using probabilistic approaches.
